# Enhancement of Perovskite Solar Cells by TiO_2_-Carbon Dot Electron Transport Film Layers

**DOI:** 10.3390/nano13010186

**Published:** 2022-12-31

**Authors:** Tamasgen Fikadu Yadeta, Kuo-Wei Huang, Toyoko Imae, Yung-Liang Tung

**Affiliations:** 1 Graduate Institute of Applied Science and Technology, National Taiwan University of Science and Technology, Taipei 10607, Taiwan; 2 Coorporation of Photovoltaic Technology Division, Green Energy & Environment Research Laboratories, Industrial Technology Research Institute (ITRI), Tainan 71150, Taiwan

**Keywords:** perovskite solar cell, mesoporous-TiO_2_, carbon dot, electron transport layer, power conversion efficiency

## Abstract

The high performance of perovskite solar cells was produced with the help of an electron transport layer (ETL) and hole transport layer. The film ETL (mesoporous (meso)-TiO_2_/carbon dot) boosted the efficiency of the perovskite solar cells. A perovskite cell was fabricated by a coating of carbon dot on a meso-TiO_2_ ETL. The fabricated meso-TiO_2_/carbon dot-based device has decreased the pin-holes of the perovskite film layer compared to the meso-TiO_2_-based device, which boosted 3% of the averaged PCE value of the devices. The UV–visible spectroscopy confirmed that the meso-TiO_2_/carbon dot ETL showed better absorbance, that is, absorbed more incident light than meso-TiO_2_ ETL to generate higher power conversion efficiency. Coating of carbon dot on meso-TiO_2_ reduced carrier recombination, and fadeaway of the perovskite film cracks. The X-ray diffraction spectra displayed the removal of the perovskite component after spin-coating of carbon dot to the meso-TiO_2_ ETL, indicating that the suppression of non-radiative recombination improves the device performance compared to meso-TiO_2_ ETL. The stability after four weeks on the performance of the device was improved to be 92% by depositing carbon dot on meso-TiO_2_ ETL compared to the meso-TiO_2_ ETL-based device (82%). Thus, the high-quality perovskite cell was fabricated by coating carbon dot on a meso-TiO_2_ ETL, because the electron transport between ETL and perovskite film layer was improved by the injection of electrons from carbon dot.

## 1. Introduction

In the last few decades, photovoltaic cells of organic–inorganic hybrid lead halide perovskites and similar materials have attracted a lot of attention from researchers. Since the first record of perovskite-based solar cell efficiency [1], organometal halide perovskites become promising materials for numerous optoelectronic and photonic devices containing solar cells, light-emitting diodes, and photoanodes [2]. Active research based on organic–inorganic hybrids has been focused on perovskite solar cells because of the recent achievements in increasing their power conversion efficiency. The energy conversion efficiency has been quickly improving with contemporary figures ranging from 15% to 25% [3,4,5,6]. This increase in performance was obtained by improving the device architecture [7,8], the layers of perovskite [9], and the levels of carrier transportation and extraction [10].

In the structure of perovskite solar cells, the electron and hole transport layers (ETL and HTL) are the key compositions to control the device performance. In particularly, the ETL is one of the most essential components in the construction of perovskite solar cells since it transports and extracts photogenerated electrons, and instantly blocks the holes. Many researchers have reported on metal oxide ETL materials such as ZnO, SnO_2_, mesoporous-TiO_2_ (meso-TiO_2_), and Al_2_O_3_ [11,12,13,14]. Among the mentioned metal oxides, meso-TiO_2_ is the most often utilized ETL in high-efficiency perovskite solar cells, because of its appropriate conduction band, thermally and chemically stabile, facial and changeable deposition techniques, environmental friendliness, easy fabrication, and a lot of attention in dye-sensitized solar cells [11,15]. Perovskite solar cells with an n-type, perovskite, and p-type architecture suppressed the charge carrier recombination and enhanced the perovskite solar cell efficiency [16]. The meso-TiO_2_ perovskite solar cells are often combined with a compact (c)-TiO_2_ ETL that successfully transports the electrons created by the perovskite layer to the external circuit and simultaneously acts as a blocking layer to prevent photogenerated holes entering into the anode, inhibiting charge recombination [17]. Nevertheless, there is still some recombination of charges between meso-TiO_2_ and perovskite layers, leading to some surface issues [18].

Carbon dot (Cdot) is an intriguing class of recently found carbon nanomaterials that consists of discrete, quasi-spherical nanoparticles with a size less than 3 nm [19]. Cdot has sparked great interest due to its low toxicity, less size, low cost, large specific surface area, and photobleaching resistance [20]. In photovoltaic devices, Cdot is effective and enhanced the performance of the devices when they were added to photovoltaic materials [19,21,22,23], despite low conductivity [24]. The photo-induced charge separation of Cdot is a crucial impact on the properties of electron carriers. Additionally, Cdot plays an important role in electron transmission and reducing the combination of electrons and redox couples. Nitrogen-rich Cdot also intensifies the fluorescence [25]. To improve the charge recombination between meso-TiO_2_ and the perovskite layer, the performance of perovskite solar cells may play a significant role as well as dye-sensitized solar cells [21,22,23].

In this study, Cdot is coated on meso-TiO_2_ nanoparticles to build a film ETL for effective perovskite solar cells. A conventional perovskite solar cell is layered by fluorine-doped tin oxide (FTO)/glass cathode, c-TiO_2_, meso-TiO_2_ ETL, (FA_0.8_MA_0.15_Cs_0.05_)Pb (I_0.85_Br_0.15_) perovskite, Spiro-OMeTAD HTL, and gold anode, where FA is the formamidinium ion (HC(NH_2_)_2_^+^) and MA is the methylammonium ion (CH_3_NH_3_^+^). The coating of an appropriate amount of Cdot on meso-TiO_2_ as the ETL may passivate crystal defects and enhance the crystallinity and the power conversion efficiency (PCE) of perovskite solar cells. Then, the Cdot-including ETLs could be more performant through interface engineering, such as an increase in carrier mobility, a reduction in charge accumulation, and efficient collection of the electrons.

## 2. Experimental Section

### 2.1. Materials and Methods

Used materials were meso-TiO_2_ paste (30NR-D; Greatcell Solar, Lonsdale, Australia), isopropanol (99.8%; Acros organics, Doral, NW, USA), ethylenediamine (99%; Acros Organics, Doral, NW, USA), citric acid anhydrous (99%; Acros Organics, Doral, NW, USA), ethyl acetate (Duksan pure chemicals, Ansan, South Korea), formamidinium iodide (FAI; Greatcell Solar, Lonsdale, Australia), methylammonium bromide (MABr; Greatcell Solar, Lonsdale, Australia), lead bromide (PbBr_2_, 99%; Tokyo chemical industry, Fukaya, Japan), lead iodide (PbI_2,_ 99.999%; Tokyo chemical industry, Fukaya, Japan), dimethylformamide (DMF, >99.8%; Acros Organics, Doral, NW, USA) and dimethyl sulfoxide (DMSO, 99.9%; J.T. Baker, Phillipsburg, NJ, USA), cesium iodide (CsI, 99.999%; Greatcell Solar, Lonsdale, Australia), chlorobenzene (99.5%; J.T. Baker, Phillipsburg, NJ, USA), Spiro-OMeTAD (LT-S922; Lumtec, New Taipei, Taiwan), bis (trifluoromethane)sulfonamide lithium salt (Li-TFSI, > 98%; Alfa, Tewksbury, NJ, USA), 4-tert-butylpyridine (tBP, 96%; Sigma, Taichung, Taiwan) and gold (Newcrest, Melbourne, Australia). The chemicals were used as they were received without additional purification.

Characterizations were performed using a field emission scanning electron microscope (FESEM, ZEISS supra 55VP, Freising, Germany), an ultraviolet (UV)–visible absorption spectroscopy (JASCOV-570, Kyoto, Japan), and a solar simulator (SS-F5-3A; Enlitech, Freiburg, Germany) under 1-sun AM 1.5G illumination (100 mW/cm^2^, 300-Watt xenon lamp; Enli Technology Co., Ltd., Freiburg, Germany). The light intensity of simulator was adjusted using a calibrated Si (silicon) solar cell, the solar cell spectral response measurement was performed to obtain the incident photon to converted electron (IPCE) spectra (QE-R; Enli Technology Co. Ltd., Freiburg, Germany), The electrochemical impedance spectroscopy (EIS) measurement was performed with an alternate current (AC) amplitude of 10 mV at a frequency spectrum from 100 mHz to 100 kHz by applying 0.5 V bias voltage in an aqueous Fe(CN)_6_^3−^ electrolyte solution on a Zahner Zennium workstation utilizing a standard three-electrode setup, which included the perovskite-based device for the working electrode, an Ag/AgCl reference electrode, and a Pt wire as a counter electrode. Time-resolved photoluminescence (PL) decay was measured using a PL spectroscopy (FluoTime 300; PicoQuant, Berlin, Germany), X-ray diffraction (XRD), and X-ray photoelectron spectroscopy (XPS) measurements were carried out using a Bruker D-8 Advance diffractometer with Cu Kα X-ray radiation (Taylor, Michigan, USA), and an X-ray photoelectron spectrometer (VG Scientific ESCALAB 250, Birmingham, UK).

### 2.2. Fabrication of TiO_2_ Blocking Layer, C-Dot, and Perovskite Solar Cells

Cdot is the same product characterized previously [23], which was synthesized using a bottom-up hydrothermal technique. Briefly, citric acid (1.0 g) and ethylenediamine (EDA, 0.3 g) (CA: EDA = 1:1 mol ratio) were dissolved in water (10 mL). The solution was transferred to a Teflon-lined autoclave and heated at 230 °C for 5 h. The product solution was transparent and dark brown. The FTO/glass substrate (8–10 Ω per square cm; Pilkington, Taiwan) was cleaned consecutively with detergent, water, acetone, and ethyl alcohol for 10 min each in an ultrasonic bath. The pre-cleaned FTO substrate was dried and immersed in aqueous 0.04 M TiCl_4_ solution at 80 °C for 30 min. The substrate was washed with water before being treated with TiCl_4_ for the required number of cycles. After following a cycle of the immersion process, the c-TiO_2_ coated substrate was washed with water and dried with an air drier. The meso-TiO_2_ paste (1:7 mass ratio of meso-TiO_2_ to ethyl alcohol) was spin-coated at 3000 rpm for 20 sec on the c-TiO_2_ covered substrate. The meso-TiO_2_ layer was dried for 10 min at 120 °C, annealed for 1 h at 400 °C, and cooled down to room temperature. An appropriate amount of Cdot dispersed in isopropanol was spin-coated on meso-TiO_2_ and dried at 120 °C for 10 min.

**Scheme 1:**
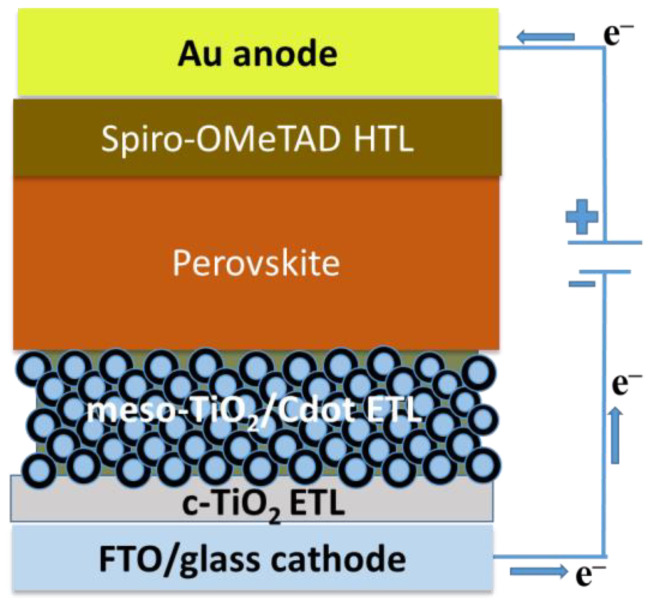
Scheme of a perovskite solar cell device with a structure of FTO/c-TiO_2_/meso-TiO_2_/Cdot/(FA_0.8_MA_0.15_Cs_0.05_)Pb(I_0.85_Br_0.15_)perovskite/Spiro-OMeTAD/Au.

The perovskite precursors were cautiously produced inside an N_2_-filled glovebox with ppm levels of oxygen and moisture [26]. The (FA_0.8_MA_0.15_Cs_0.05_)Pb(I_0.85_Br_0.15_)_3_ precursor solution was developed by mixing FA, PbBr_2_, MABr, PbI_2_, and CsI solution (35 μL, 1.35 M dissolved in DMSO) in a co-solvent of DMF/DMSO (1:4 *v*/*v*) and by heating for 60 min at 70 °C in a glovebox filled with nitrogen. The prepared perovskite precursor solution was deposited on the meso-TiO_2_/Cdot-coated substrate at two steps of spin-coating at 2000 rpm for 10 s and 6000 rpm for 30 s. Ethyl acetate (300 µL) was carefully dripped onto the substrate to produce a perovskite film layer during final 20 s of the second spin coating. The perovskite film layer was dried for 1 h at 100 °C on a hot plate and cooled down to room temperature. The hole transport materials were deposited on perovskite film layer: 72.3 mg of spiro-OMeTAD with additives of 17.5 μL of Li-TFSI (26 mg in 100 μL acetonitrile) and 28.8 μL tBp in 1 mL chlorobenzene was deposited on the top of perovskite film at 3000 rpm for the 30 s in a dry-air glovebox. Lastly, as the top electrode for perovskite solar cells, 80 nm gold electrode was evaporated on the spiro-OMeTAD layer. Scheme 1 shows the structural design of the produced FTO/c-TiO_2_/meso-TiO_2_/Cdot/(FA_0.8_MA_0.15_Cs_0.05_)Pb(I_0.85_Br_0.15_)/Spiro-OMeTAD/Au.

## 3. Results and Discussion

**Figure 1:**
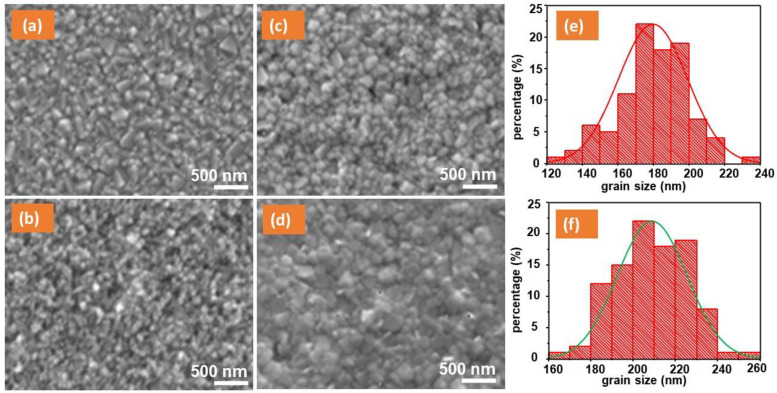
SEM images of: (**a**) bare FTO/glass; (**b**) FTO/c-TiO_2_/meso-TiO_2_; (**c**) FTO/c-TiO_2_/meso-TiO_2_/perovskite; and (**d**) FTO/c-TiO_2_/meso-TiO_2_/Cdot/perovskite, Grain-size histograms on perovskite surface of (**e**) FTO/c-TiO_2_/meso-TiO_2_/perovskite and (**f**) FTO/c-TiO_2_/meso-TiO_2_/Cdot/perovskite.

A perovskite solar cell device was created based on (FA_0.8_MA_0.15_Cs_0.05_)Pb(I_0.85_Br_0.15_) perovskite layer sandwiched between c-TiO_2_/meso-TiO_2_/Cdot ETL and Spiro-OMeTAD, HTL. Then, the efficiency of Cdot added in an ETL was examined. The SEM image of the FTO glass substrate is shown in Figure 1a. The FTO surface has a rough texture and grains randomly distribute. Figure 1b is a meso-TiO_2_ surface that was spin-coated on FTO and calcined at 450 °C for 1 h. Inhomogeneous distribution of grains may result in an aggregation and substantially affect the efficiency of device [26,27]. Figure 1c shows that large perovskite grains spin-coated on FTO/meso-TiO_2_ including Cdot also displayed a similar texture (Figure 1d).

As seen in Figure 1e,f, the grain size distribution (209 ± 16 nm) on FTO/c-TiO_2_/meso-TiO_2_/Cdot/perovskite was greater than that (180 ± 19 nm) of FTO/c-TiO_2_/meso-TiO_2_/perovskite. According to a report, the device with a larger grain size has a better performance [28]. This implies a high contribution of Cdot in ETL to make a better grain size and better interface with perovskite. Meso-TiO_2_ is employed as an electron transport medium, and Cdot is spin-coated on it to facilitate more electron injections and to improve the interface between meso-TiO_2_ and perovskite. Since the meso-TiO_2_-based devices have demonstrated a good performance in prior studies, but there are still surface concerns between the electron transport medium and the perovskite interface, it was improved by the doping of Cdot to meso-TiO_2_ ETL. In addition, the mechanism of action of the Cdot to boost the efficiency of the device was investigated.

**Figure 2:**
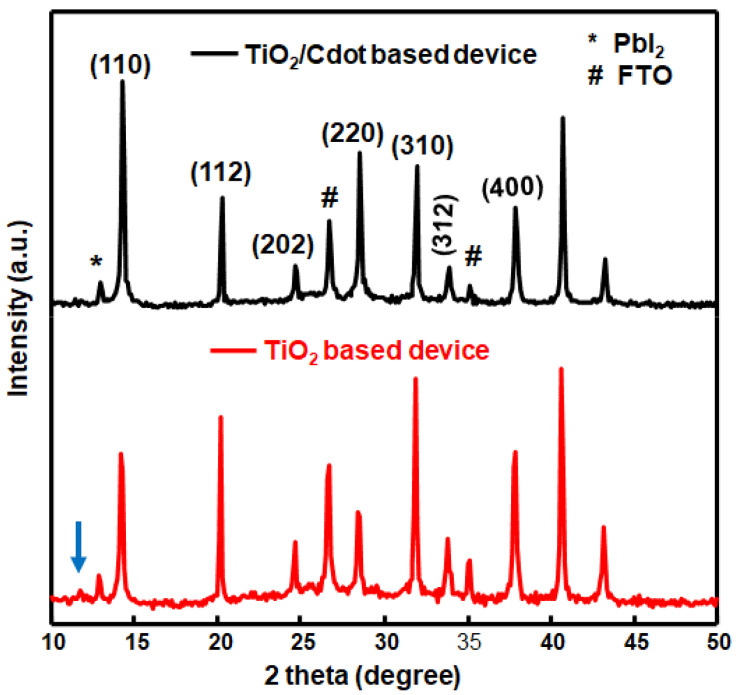
XRD spectra of meso-TiO_2_ and meso-TiO_2_/Cdot-based devices. A peak marked by a blue arrow is attributed to a photoinactive phase of δ-FAPbI_3_.

The XRD patterns of the FTO/c-TiO_2_/meso-TiO_2_/perovskite with Cdot and without Cdot are shown in Figure 2. The prominent peaks at 14.22°, 20.16°, 24.70°, 28.54°, 31.94°, 33.87°, and 37.88° are assigned to the (110), (112), (202), (220), (310), (312), and (400) planes of perovskite with and without c-TiO_2_. The peaks at 11.69° and 12.83° are ascribed to the photoinactive phases of δ-FAPbI_3_ and PbI_2_, respectively. The modest excess of PbI_2_ in perovskite can prevent the nonradiative charge-carrier recombination, resulting in excellent external electroluminescence quantum efficiency [26]. The spin-coating of Cdot has changed the perovskite crystal structure which was verified by an XRD measurement. Although a weak δ-FAPbI_3_ peak was presented at 11.69° diffraction angle in FTO/c-TiO_2_/meso-TiO_2_/perovskite (see an arrow in Figure 2), it disappeared in FTO/c-TiO_2_/meso-TiO_2_/Cdot/perovskite. Thus, the addition of Cdot to the perovskite solar cell reduced the density of defect δ-FAPbI_3,_ which suppresses the non-radiative recombination of charge carriers [29] and improves the device performance. Strong and narrow signals of XRD reveal the substantial crystalline structure of the perovskite device.

**Figure 3:**
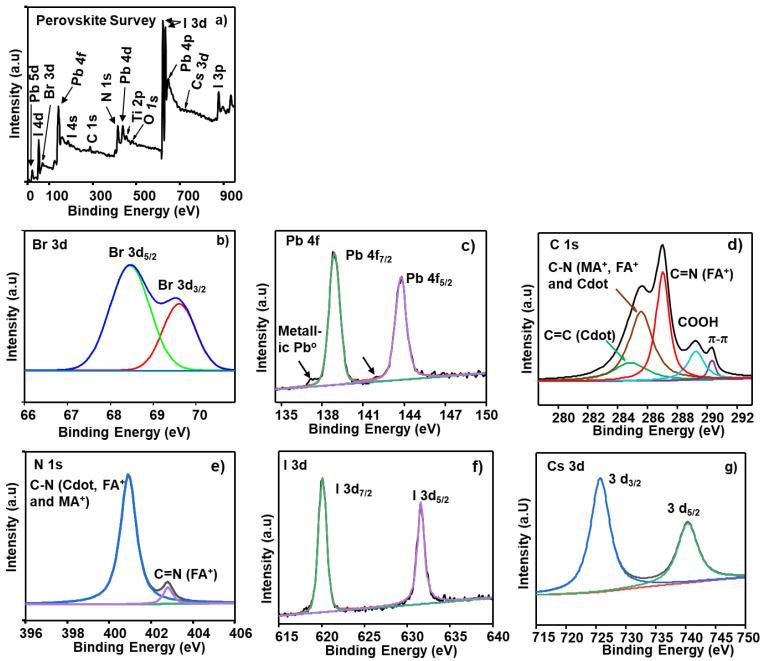
XPS spectra of (**a**) Survey; (**b**) Br 3d; (**c**) Pb 4f; (**d**) C 1s; (**e**) N 1s; (**f**) I 3d; and (**g**) Cs 3d of FTO/c-TiO_2_/meso-TiO_2_/Cdot/perovskite.

A whole XPS survey scan of an FTO/c-TiO_2_/meso-TiO_2_/Cdot/(FA_0.8_MA_0.15_Cs_0.05_)Pb(I_0.85_Br_0.15_)perovskite is shown in Figure 3a. The distinctive strong peaks of carbon, nitrogen, iodine, and lead are ascribed to perovskite, but the peaks of bromine and cesium are weak because their contents in perovskite are low compared with other elements. Moreover, peaks of oxygen and titanium are also weak because the TiO_2_ layer is under the thick perovskite layer (~680 nm).

Figure 3b–g show XPS spectra of elements and their deconvolution results, and the numerical values of binding energies and area intensities are listed in Table 1. The XPS of Pb 4f spectrum exhibits spin orbit splitting peaks of Pb 4f_7/2_ and Pb 4f_5/2_ at 138.9 and 143.8 eV, respectively, and the lead ion keeps in the state of Pb^2+^. Two peaks at 137.2 and 141.9 eV due to the presence of metallic Pb^0^ [30] are almost invisible. The existence of these peaks is undesirable, because metallic Pb^0^ behaves as non-radiative recombination centers and makes it difficult to transmit and collect photogenerated charge carriers [31]. Peaks of I (3d_3/2_ and 3d_5/2_) and Br (3d_3/2_ and 3d_5/2_) were observed at 620.1, 631.6, and 68.5, 69.6 eV, respectively [32], and Cs 3d peaks were split into 3d_3/2_ and 3d_5/2_. These peaks were shifted toward a higher binding energy at 740.3 and 725.7 eV than values at 738.2 and 724.3 eV, respectively, for the device without Cdot [33]. Thus, this shift may consider the effect of addition of Cdot, although Pb, I, and Br elements were not affected by the addition of Cdot.

Peak positions in the N1s spectra of FA^+^ and MA^+^ cations in perovskite have been reported as being located at 400.3 and 402.0 eV for C–N and C=N bonds, respectively [34]. However, after the addition of Cdot to the TiO_2_-based perovskite device, their peaks shifted to the higher binding energy of 400.9 and 402.8 eV, respectively. A C–N bond (400.7 eV) of Cdot may overlap to the binding energy at a lower energy bond but the deconvolution was difficult [23,34].

C 1s had five deconvoluted peaks of two main peaks at 285.6 and 287.1 eV and three weak peaks at 284.7, 289.2, and 290.3 eV. Strong bands are assigned to C–N bond of FA and MA and C=N bond of FA. Weak bands can be contributed by Cdot and represented bonds of C=C (aromatic), C=O (carboxylic) [35,36] and graphitic π–π* satellite [37], respectively, although the C–N bond in Cdot was overlapped with the C–N bond of FA^+^ and MA^+^ [38]. The result shows that Cdot coated on meso-TiO_2_ and neighbored to perovskite was detected on XPS spectra.

**Figure 4:**
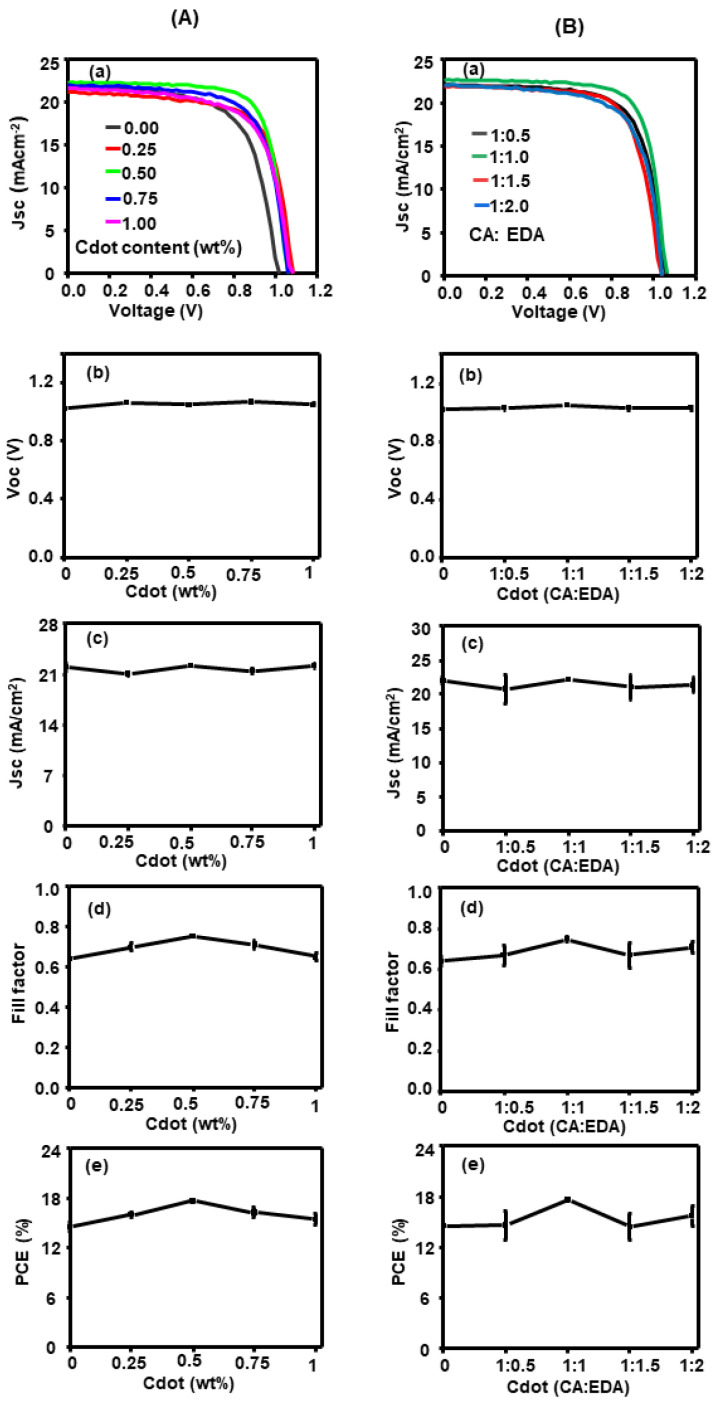

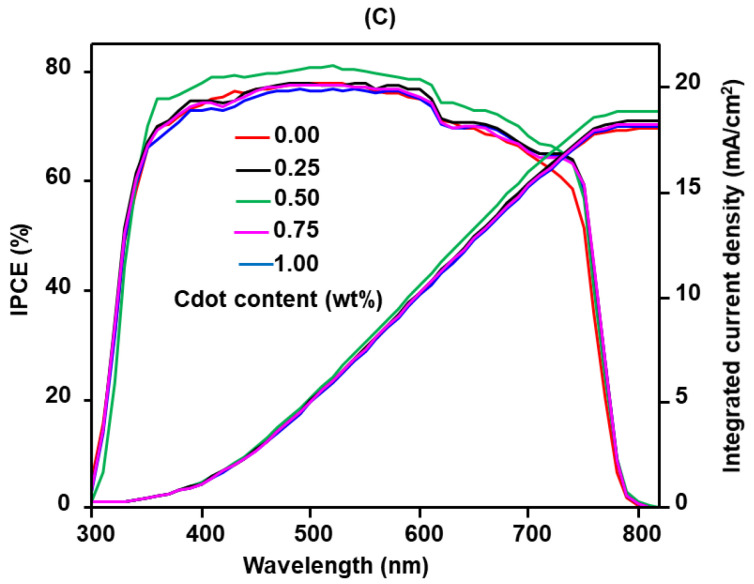
Current density–voltage curves of (**A**) FTO/c-TiO_2_/meso-TiO_2_/Cdot (1:1)/perovskite/spiro-OMeTAD/Au solar cells at different contents of Cdot and (**B**) FTO/c-TiO_2_/meso-TiO_2_/Cdot (1.0 wt%)/perovskite/spiro-OMeTAD/Au solar cells at different CA: EDA mol ratios of Cdot. (**a**) J–V curve; (**b**) V_oc_; (**c**) J_sc_; (**d**) fill factor; and (**e**) PCE. (**C**) IPCE spectra of perovskite solar cells at different contents of Cdot.

Figure 4A(a) indicates the current density–voltage curves of meso-TiO_2_-based and meso-TiO_2_/Cdot-based devices, which were analyzed under simulated one sun. A meso-TiO_2_/Cdot ETL-based device showed an excellent averaged PCE of 17.58% with a short circuit current density (J_sc_) of 22.19 mA/cm^2^, open circuit voltage (V_oc_) = 1.05 V, and fill factor (FF) = 0.75, exceeding the meso-TiO_2_ devices, which is a PCE of 14.52% with J_sc_ of 22.02 mA/cm^2^, V_oc_ = 1.02 V, and FF = 0.64. The effect of Cdot was shown on the plotted graphs. The highest current density was achieved by the spin-coating of meso-TiO_2_/Cdot (0.5 wt%). Figure 4A(b–e) plot the calculated parameters as a function of Cdot content. Although V_oc_ and J_sc_ do not change, FF had a maximum at meso-TiO_2_/Cdot (0.5 wt%). The increment of FF influenced the enhancement of the PCE of the perovskite device. Figure 4B indicates the current density–voltage curves of meso-TiO_2_/Cdot (0.5 wt%)-based device at different CA: EDA mole ratios of Cdot and the plots of obtained parameters as a function of CA:EDA mol ratio. The cell at CA:EDA = 1:1 had the highest FF value among different CA:EDA (=1:0.5, 1:1, 1:1.5 and 1:2) mol ratios, although J_sc_ and V_oc_ did not show a variation. In the same manner, PCE increased at Cdot (1:1) mole ratio, indicating the increased performance of the device.

The meso-TiO_2_/Cdot ETL plays a superior function to enhance the PCE of the perovskite device, since the electrical parameters of the meso-TiO_2_/Cdot-based device have higher performances than the meso-TiO_2_-based devices. Thus, the excellent reliability of the Cdot was demonstrated. As seen in Figure 4C, IPCE was also highest at meso-TiO_2_/Cdot (0.5 wt%). The increased IPCE value can be thought to be due to greater light absorption and increased photon-to-electricity conversion capabilities. The deposition of Cdot on meso-TiO_2_ improves the surface issues between the meso-TiO_2_ and perovskite film layer [39]. Because Cdot as a sensitizer injects electrons [40], meso-TiO_2_/Cdot can enhance the performance of the perovskite film layer and simultaneously act as a barrier for the charge recombination at its interface with the perovskite film layer. The surface improvement of the meso-TiO_2_/Cdot-based perovskite solar cells raises great performance. Cdot acts as a bridge between meso-TiO_2_ and perovskite, encouraging the electron injection capacity of the perovskite and meso-TiO_2_ layers. As a result, the performance of a device can be improved [41].

**Figure 5:**
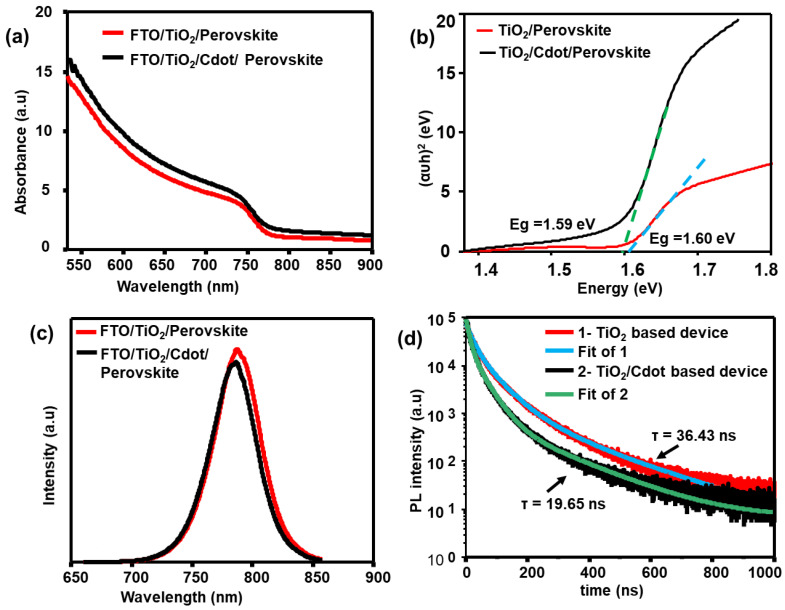
Graph of (**a**) UV–visible absorbance spectra; (**b**) Tauc plots; (**c**) PL spectra; and (**d**) time-resolved photoluminescence plots and fitting curves of meso-TiO_2_ and meso-TiO_2_/Cdot based devices.

Figure 5a shows the UV-visible absorption spectra of perovskite layer on meso-TiO_2_ and meso-TiO_2_/Cdot-based devices. The absorbance of both spectra decreased with increasing wavelength till 750 nm and kept constant above 800 nm, but the absorbance was always stronger for meso-TiO_2_/Cdot-based perovskite film layer than the meso-TiO_2_-based perovskite film layer. It should be noted that no 588 nm band of PbI_2_ was present [42], suggesting the better photovoltaic performance [43]. The optical bandgaps calculated from the Tauc plot (Figure 5b) for layers of meso-TiO_2_-based and meso-TiO_2_/Cdot-based devices were 1.59 and 1.60 eV, respectively, indicating almost the same bandgap between both devices.

The steady-state and time-resolved photoluminescence (PL) spectra of perovskite solar cells with and without Cdot were also measured. Figure 5c shows that the steady-state PL of the FTO/c-TiO_2_/meso-TiO_2_/perovskite cell decreased in intensity and blue-shifted, when Cdot was coated. The decreased intensity means that there is a charge transfer from perovskite to Cdot [44] and the photo-induced carriers may be retrieved from perovskite layers to electrode layers by minimizing carrier recombination. The blue-shift indicates lower thermal losses or the presence of fewer energy levels between the conduction band and valence band in comparison to meso-TiO_2_-based devices [45].

In the time-resolved PL behavior (Figure 5d), the PL intensity decreased with time and the decay was faster for the device with Cdot than without Cdot. The PL lifetime was calculated by fitting the observed PL spectra with an exponential decay equation, $I\left(t\right)={\mathrm{Ae}}^{-t/\tau }$ [44] as shown in Figure 5d in a time frame of the first 1000 ns. The calculated PL lifetimes of the FTO/c-TiO_2_/meso-TiO_2_/perovskite-based and FTO/c-TiO_2_/meso-TiO_2_/Cdot/perovskite-based devices were 36.4 ns and 19.7 ns, respectively. As a result, the fast PL lifetime indicates the faster charge transfer and then the suppressing charge carrier recombination. Then, the addition of Cdot to perovskite solar cells has a great contribution to suppress the charge recombination. For pristine thin-film perovskite, a quick decay lifetime (12.9 ns) has been reported, but there is also a report of a slow decay lifetime of 104.3 ns [46]. The results demonstrate that the decay process is influenced by some factors including the charge transfer.

**Figure 6:**
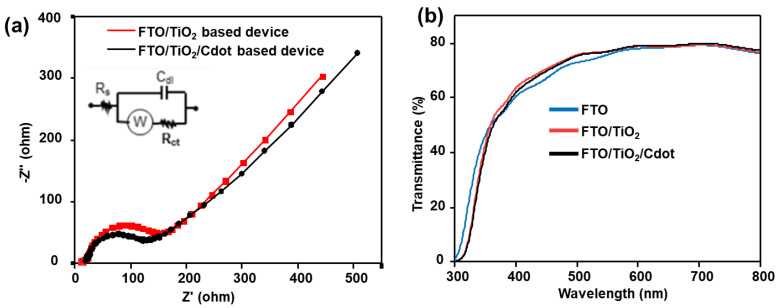
(**a**) EIS spectra of meso-TiO_2_- and meso-TiO_2_/Cdot-based devices, and (**b**) transmittance spectra of FTO, meso-TiO_2_, and meso-TiO_2_/Cdot based devices.

The Nyquist plots of EIS were measured for working electrodes of meso-TiO_2_- and meso-TiO_2_/Cdot-based perovskite solar cells and their quantitative analysis was conducted by fitting an analogous circuit (Figure 6a). While the series resistance (Rs) is mostly related to the resistance of the FTO electrode [47,48], the charge transfer resistance (R_ct_) causes the size of semicircle arc in the indicated frequency range [48].

The R_ct_ of the meso-TiO_2_/Cdot- and meso-TiO_2_-based devices were 65.9 and 77.5 Ω, respectively: the R_ct_ of the meso-TiO_2_/Cdot-based device is less than the meso-TiO_2_-based devices. This suggests that the incorporation of Cdot in ETL layers can greatly increase the preferable contact between the active layer and the electrodes by lowering the resistance of the charge transfer process. The improved resistance suggests a decreased charge recombination at the meso-TiO_2_/Cdot/perovskite contact. For this reason, it is plausible that a meso-TiO_2_/Cdot-based cell has a greater PCE than a meso-TiO_2_-based cell. Figure 6b, depicts the transmittance spectra of bare FTO glass, FTO/meso-TiO_2_, and FTO/meso-TiO_2_/Cdot substrates. The transmittance of the FTO/meso-TiO_2_ and FTO/meso-TiO_2_/Cdot was slightly greater than that of the bare FTO at a visible light region of 400–600 nm. The similar transmittance of FTO/meso-TiO_2_/Cdot to FTO/meso-TiO_2_ is due to the non-influence on the light adsorption at ETL even after coating by Cdot.

**Figure 7:**
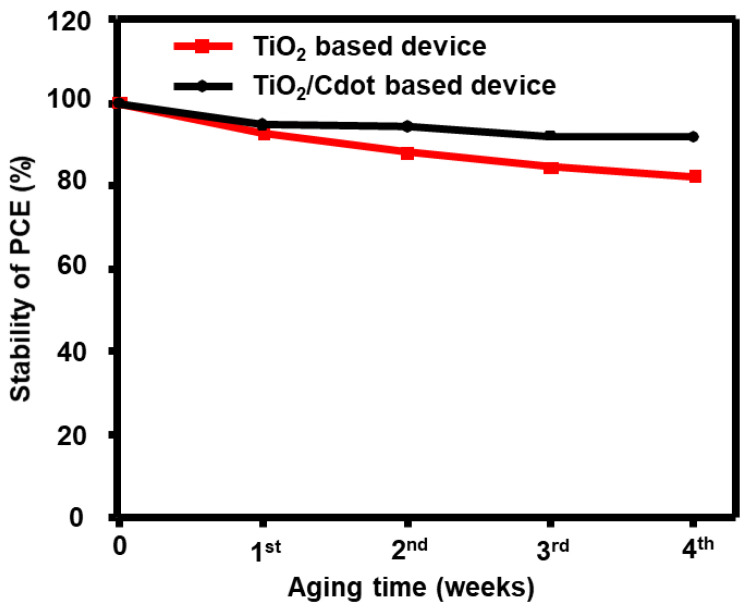
The stability of meso-TiO_2_ and meso-TiO_2_/C-dot based devices.

The stability of the perovskite solar cell is another key characteristic to consider for evaluating the device performance. The stability test of the device was performed for four weeks in air environment. As seen in Figure 7, the meso-TiO_2_/C-dot-based device exhibited superior stability of 92% against its initial value. In the same condition, the stability of the meso-TiO_2_-based device after four weeks was 82%. Improved stability is inextricably linked to improvements in the perovskite/ETL interface and perovskite film quality.

## 4. Conclusions

In conclusion, a meso-TiO_2_/Cdot ETL-based device to effect a smooth interference between ETL and perovskite films was fabricated and compared with a meso-TiO_2_ ETL-based device. A meso-TiO_2_/Cdot ETL-based device suppressed the carrier charge recombination and improved the interface with perovskite layer. The PCE of the meso-TiO_2_/C-dot (0.5 wt.%) device was enhanced to 17.58% from 14.52% of the meso-TiO_2_-based device. This enhancement inspires the prediction of the higher efficiency of the perovskite due to surface modification by Cdot and low charge transfer resistance, which increased the FF. The disappearance of δ-FAPbI_3_ from a Cdot-based device indicated that the non-radiative recombination was suppressed to contribute to solar cell performance. The contribution of the meso-TiO_2_/Cdot ETL interface with perovskite layer appeared even on the stability of the meso-TiO_2_/Cdot device (92%) better than the meso-TiO_2_-based device (82%). In the present finding, the efficiency of Cdot on the perovskite device was not the highest efficiency, but it showed the effect of Cdot as an ETL to improve the meso-TiO_2_-based devices by 21%. As a result, the Cdot is considered to be a viable dopant for improving the perovskite solar cells’ performances.

The novelty of this work is to assess the effectiveness of Cdot to advance the performance of the perovskite solar-cell device. As the meso-TiO_2_/Cdot-based device absorbs more incident light than the meso-TiO_2_-based device, the IPCE is obtaining a better performance. Thus, an improvement in the photon-to-electron conversion capability occurs after the addition of Cdot, contributing to the inhibition in the electron–hole recombination at the interface of the meso-TiO_2_/Cdot and perovskite film layer. Carbon black, fullerene, carbon nanotube, and graphene are carbon compounds that have been employed in perovskite solar cells [49]. It is noted that the hydrophobic characteristics of carbon materials make them perfect for preventing moisture-related degradation and improving the stability of perovskite solar cells. Nevertheless, a metal oxide ETL mixed with a hydrophilic graphene quantum dot achieves a high PCE value for a perovskite solar cell [28]. Thus, further investigations concerning carbon materials-encapsulated perovskite solar cells may be expected.

## Figures and Tables

**Table 1:** The XPS binding energies and area intensities of elements in Cdot-embedded perovskite solar cells.

Element	Binding Energy (eV)	Area Intensity (a.u)	Bond Assignment
Br 3d	68.5	42.9	Br^−^ 3d_5/2_
	69.6	14.9	Br^−^ 3d_3/2_
Pb 4f	138.9	955.2	Pb^2+^ 4f_7/2_
	143.8	959.1	Pb^2+^ 4f_5/2_
C 1s	284.7	47.8	C=C (Cdot)
	285.6	126.5	C–N (FA^+^, MA^+^ and Cdot)
	287.1	107.6	C=N (FA)
	289.2	37.6	COOH (Cdot)
	290.3	9.8	Graphitic π–π* satellite (Cdot)
N 1s	400.9	123.5	C–N (FA^+^, MA^+^ and Cdot)
	402.8	9.4	C=N (FA^+^)
I 3d	620.1	3440.8	I^−^ 3d_7/2_
	631.6	3515.3	I^−^ 3d_5/2_
Cs 3d	725.7	130.5	Cs^+^ 3d_3/2_
	740.3	72.0	Cs^+^ 3d_5/2_

## Data Availability

Not applicable.
